# Supplementary material for: A Hybrid Perovskite-Based Electromagnetic Wave Absorber with Enhanced Conduction Loss and Interfacial Polarization through Carbon Sphere Embedding
Source: Nanomaterials (Basel). 2024 Sep 27;14(19):1566. doi: 10.3390/nano14191566 (PMC11478511; doi:10.3390/nano14191566)
Supplement: Supplementary file 1 [file nanomaterials-14-01566-s001.zip › nanomaterials-3200810-supplementary.pdf]

Supporting information

A Hybrid Perovskite-based Electromagnetic Wave Absorber with Enhanced Conduction Loss and Interfacial Polarization through Carbon Sphere Embedding

Xuehua Lian[1†], Yao Yao[2†], Ziming Xiong[2], Yantao Duan[3], Jianbao Wang[3], Shangchen Fu[3], Yinsuo Dai[4], Wenke Zhou[3]*, and Zhi Zhang[4]*

1 Field Engineering College, Army Engineering University of PLA, Nanjing 210007, China

2 State Key Laboratory for Disaster Prevention & Mitigation of Explosion & Impact, Army Engineering University of PLA, Nanjing 210007, China

3 Electromagnetic Environmental Effects Laboratory, Army Engineering University of PLA, Nanjing 210007, China

4 Position Engineering Research Office, Army Engineering University of PLA, Nanjing 210007, China

* Correspondence: zhou.w.k@outlook.com (W.Z.); zhangnjjn@outlook.com (Z.Z.)

† These authors contributed equally to this work.

We investigate how thickness and bandwidth influence the absorption of $MAPbI_3$/CS. For a dielectric loss absorbing material, electromagnetic wave loss was dominated by charge polarization loss and conduction loss. The charge polarization loss contains three scenarios: dipole polarization, interfacial polarization and ionic relaxation polarization [1]. The Debye relaxation formula correlates with the ionic relaxation polarization and dipole polarization according to formula [2]:

$$\left(\varepsilon' - \frac{\varepsilon_s+\varepsilon_\infty}{2}\right)^2 + (\varepsilon'')^2 = \left(\frac{\varepsilon_s-\varepsilon_\infty}{2}\right)^2 \quad \text{(S1)}$$

Where ε_s is the static dielectric constant and ε_∞ is the relative dielectric constant of the high-frequency limit. According to formula (S1), we can establish a semicircle formula called the "Cole-Cole semicircle" with (0, (ε_s + ε_∞)/2) as the center and (ε_s − ε_∞)/2 as the radius of the circle. Usually, one semicircle represents one relaxation process. A larger number of the semicircles reflects a higher intensity of relaxation polarization [1,2]. As shown in Figure S1, the MS-1 and MS-3 exhibit two obvious Cole-Cole semicircles. In contrast, the MS-2 exhibits one. All the Cole-Cole semicircles are completely distributed within 2-18 GHz, indicating that the dielectric losses of the three samples mainly arise from relaxation polarization. It is particularly noted that, in the low-frequency region, the Cole-Cole semicircle of the MS-3 does not conform to the Debye relaxation law, indicating that the MS-3 also contains extra polarization processes such as interfacial and electronic polarization.

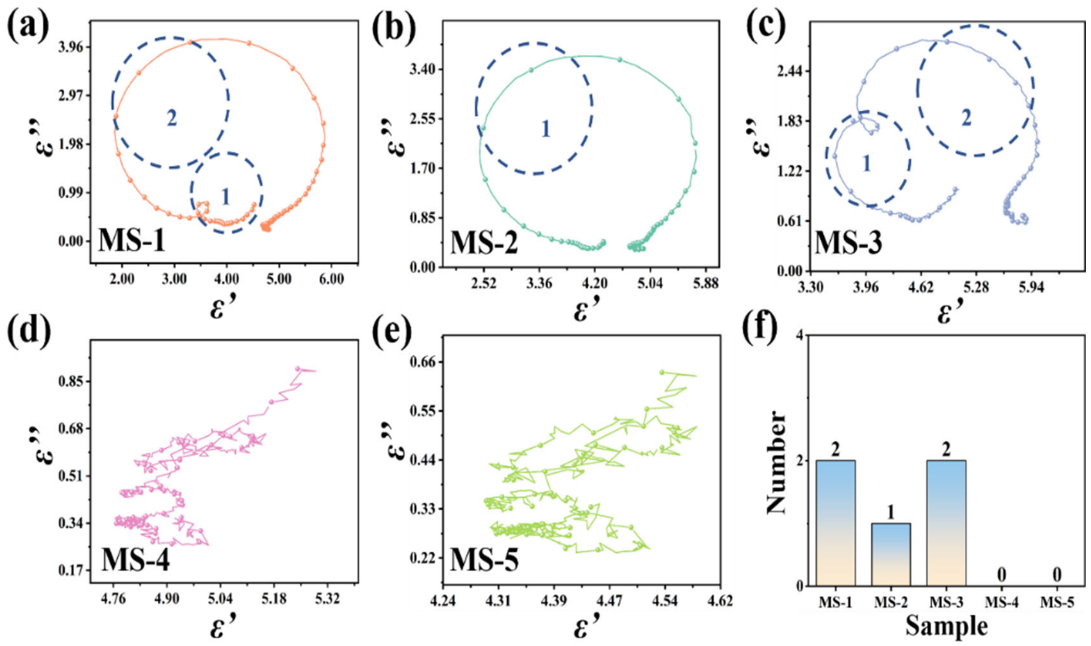

Figure S1. Cole-Cole diagram of MS-1 (a), MS-2 (b), MS-3 (c), MS-4 (d), MS-5 (e), and the number of Cole-Cole semicircles in the $MAPbI_3$/CS composites (f).

Based on the results of the RL, the impedance matching performance (IMP) performance of $MAPbI_3$/CS is shown in Figure S2 and is calculated according to formula [3]:

$$IMP = \left| \sinh^2 (Kfd) - M \right| \tag{S2}$$

$$K = \frac{4\pi\sqrt{\mu'\varepsilon'}}{c} \frac{\sin\left[\left(\delta_\varepsilon + \delta_\mu\right)/2\right]}{\cos\delta_\varepsilon \cos\delta_\mu}$$

$$M = \left(4\mu'\varepsilon'\cos\delta_\varepsilon\cos\delta_\mu\right)\left[\left(\mu'\cos\delta_\varepsilon - \varepsilon'\cos\delta_\mu\right)^2 + \left(\tan\frac{\delta_\mu - \delta_\varepsilon}{2}\right)^2 \left(\mu'\cos\delta_\varepsilon + \varepsilon'\cos\delta_\mu\right)^2\right]^{-1}$$

$$\delta_\varepsilon = \arctan\left(\frac{\varepsilon''}{\varepsilon'}\right)$$

$$\delta_\mu = \arctan\left(\frac{\mu''}{\mu'}\right)$$

IMP represents the degree of impedance matching of a material at a given electromagnetic wave frequency and thickness. The closer of the IMP value is to 0, the higher wave absorption performance exhibits. Where the IMP value within the red box is less than 0.05, and the IMP value of the area marked as white is greater than 1.5. By comparing Figure S2a–e, for MS-1, MS-2, and MS-3, the wave frequency of the IMP is less than 0.05 and appears at 12 GHz, 14 GH, and 10-14 GHz, respectively. Meanwhile, the IMP values of MS-4 and MS-5 are all greater than 0.05. The IMP results further verify the rationality of the MS reflection loss performance in Figure 6.

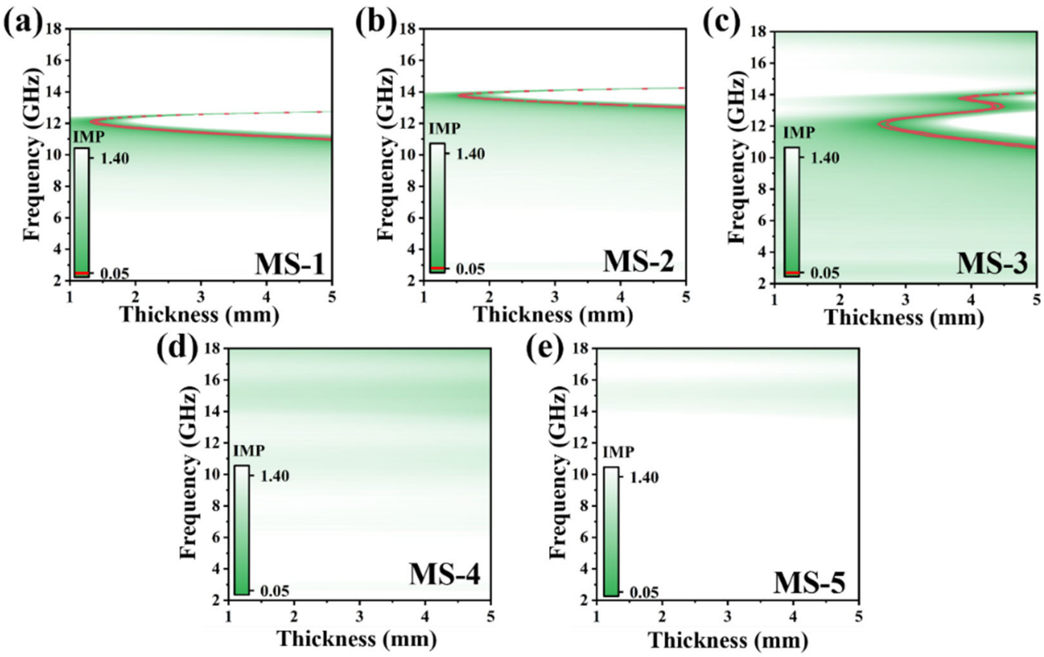

Figure S2. Impedance matching performance of MS-1 (a), MS-2 (b), MS-3 (c), MS-4 (d), MS-5 (e).

References

1. Qin, M.; Zhang, L.; Wu, H. Dielectric Loss Mechanism in Electromagnetic Wave Absorbing Materials. *Adv. Sci*. **2022**, *9*, 2105553.

2. Xie, A.; Sun, M.; Zhang, K.; Jiang, W.; Wu, F.; He, M. In situ growth of MoS_2 nanosheets on reduced graphene oxide (RGO) surfaces: Interfacial enhancement of absorbing performance against electromagnetic pollution. *Phys. Chem. Chem. Phys.* **2016**, *18*, 24931-24936.

3. Song, L.; Duan, Y.; Liu, J.; Pang, H. Transformation between nanosheets and nanowires structure in MnO_2 upon providing Co^{2+} ions and applications for microwave absorption. *Nano Res.* **2020**, *13*, 95-104.
